# Generation of a Porcine Cell Line Stably Expressing Pig TMPRSS2 for Efficient Isolation of Swine Influenza Virus

**DOI:** 10.3390/pathogens13010018

**Published:** 2023-12-24

**Authors:** Yuri L Tanaka, Maya Shofa, Erika P Butlertanaka, Ahmad Massoud Niazi, Takuya Hirai, Hirohisa Mekata, Akatsuki Saito

**Affiliations:** 1 Laboratory of Veterinary Microbiology, Department of Veterinary Science, Faculty of Agriculture, University of Miyazaki, Miyazaki 8892192, Japan; 2 Graduate School of Medicine and Veterinary Medicine, University of Miyazaki, Miyazaki 8891692, Japan; 3 Laboratory of Veterinary Pathology, Department of Veterinary Science, Faculty of Agriculture, University of Miyazaki, Miyazaki 8892192, Japan; 4 Center for Animal Disease Control, University of Miyazaki, Miyazaki 8892192, Japan

**Keywords:** influenza virus, isolation and propagation, PK-15 cell, TMPRSS2

## Abstract

Pigs are important animals for meat production but can carry several zoonotic diseases, including the Japanese encephalitis virus, Nipah virus, and influenza viruses. Several *Orthomyxoviridae* and *Coronavirinae* respiratory viruses require cleavage of envelope proteins to acquire viral infectivity and consequently, need a host protease or the addition of exogenous trypsin for efficient propagation. Host TMPRSS2 is a key protease responsible for viral cleavage. Stable expression of human TMPRSS2 in African green monkey-derived Vero cells can enhance the porcine epidemic diarrhea virus. However, considering the narrow host tropism of viruses, a porcine cell line expressing pig TMPRSS2 could be optimal for replicating pig-derived viruses. Herein, we generated and evaluated a pig-derived PK-15 cell line stably expressing pig TMPRSS2. This cell line markedly (>1000-fold) and specifically enhanced the growth of influenza viruses. Furthermore, we demonstrated the usefulness of a PK-15 cell line lacking the *Stat2* gene with a stable expression of pig TMPRSS2 for efficient virus isolation from clinical samples in the presence of type I interferons. Therefore, PK-15 cells expressing pig TMPRSS2 could be a valuable and promising tool for virus isolation, vaccine production, and virological studies of TMPRSS2-dependent viruses.

## 1. Introduction

Pigs can host various viral infections that can cause significant economic damage to the swine industry [1,2]. Pigs can also be a host for several zoonotic viral diseases, including influenza [2], Nipah virus disease [3], and Japanese encephalitis [4]. In particular, pigs can serve as an intermediate host for influenza viruses between birds and humans [5]. To infect cells, human influenza viruses recognize sialic acid (human receptor) linked to galactose (α2-6), while avian influenza viruses recognize sialic acid linked to α2-3 (avian receptor). Previous studies demonstrated that pigs express both human and avian receptors for influenza [6,7]. Therefore, pigs can be infected with both human and avian influenza A viruses, leading to gene reassortment and the emergence of new strains of influenza that are more likely to spread in humans [8,9]. The H1N1 influenza A virus that emerged in 2009 to cause a worldwide pandemic was a porcine-derived influenza virus that spread to humans. Thus, pigs can be an essential host in the spread of infection.

Several respiratory viruses, including *Orthomyxoviridae* and *Coronavirinae*, require cleavage of viral membrane fusion proteins by host proteases to achieve optimal infectivity [7], and trypsin must be added to the culture medium to propagate these viruses. However, since the effect of trypsin is weakened by a culture medium containing high concentrations of fetal bovine serum (FBS), these concentrations must be reduced. This makes subsequent virus isolation and propagation from animal specimens with respiratory symptoms more laborious and complex.

During infection with the influenza virus, viral hemagglutinin protein (HA) is responsible for the fusion between viral and endosomal membranes during virus entry into the cell. HA is synthesized as the fusion-incompetent precursor protein HA0; thus, it requires cleavage by a host cell protease into the subunits HA1 and HA2 to obtain infectivity. TMPRSS2 is a key protease that activates HA in the respiratory tract [8]. In humans, TMPRSS2 is expressed in a wide range of tissues, including the lung, intestine, and prostate [9]. However, most cell lines do not express TMPRSS2. Previous studies established a Vero cell line with stable expression of human TMPRSS2 [10]. Human metapneumovirus and SARS-CoV-2 can reportedly be isolated and propagated more efficiently in the Vero/TMPRSS2 cell line than in normal Vero cells [10,11]. Furthermore, the isolation efficiency of the porcine epidemic diarrhea virus was also improved using Vero/TMPRSS2 cells [12]. However, the homology of TMPRSS2 sequences between humans and pigs is low, limiting the use of cells expressing human TMPRSS2 for virus isolation.

Furthermore, during the step of viral isolation, contamination of type I interferons (IFNs) or IFN-inducible substances such as double-stranded RNA or lipopolysaccharides can exert antiviral effects in the target cells. In this cascade, both the cellular surface IFN alpha and beta receptor subunit 1 (IFNAR1) and intracellular STAT2 play a critical role [13]. We recently generated a pig-derived PK-15 cell line lacking *Ifnar1* or *Stat2* genes to overcome this limitation [14].

Herein, we aimed to establish a PK-15 cell line stably expressing pig TMPRSS2 (PK-15/TMPRSS2 cells) to improve the isolation efficiency of respiratory tract-derived viruses that utilize TMPRSS2 for optimal replication. A viral replication assay showed that PK-15/TMPRSS2 cells enhanced the replication of the H1N1 influenza virus >1000-fold more than that of normal PK-15 cells. Furthermore, we demonstrated the usefulness of a PK-15 cell line lacking the *Stat2* gene with a stable expression of pig TMPRSS2. Finally, we demonstrated that nafamostat mesylate inhibited viral replication in PK-15/TMPRSS2 cells, suggesting the specific enhancement by pig TMPRSS2. These findings indicate that PK-15/TMPRSS2 cells are a promising tool for isolating and propagating TMPRSS2-dependent viruses, including *Orthomyxoviridae* and *Coronavirinae* viruses, from respiratory or digestive organs.

## 2. Materials and Methods

### 2.1. Plasmids

The plasmids psPAX2-IN/HiBiT and pWPI-Luc2 were kind gifts from Dr. Kenzo Tokunaga [15]. pMD2.G was a gift from Dr. Didier Trono (Cat# 12259; http://n2t.net/addgene:12259, accessed on 26 November 2023; RRID: Addgene_12259).

### 2.2. Cell Culture

Lenti-X 293T (TaKaRa, Kusatsu, Japan, Cat# Z2180N), PK-15 (Japanese Collection of Research Bioresources Cell Bank (JCRB), Ibaraki, Japan, Cat# JCRB9040), and PK-15 (*Stat2* k/o) cells [14] were cultured in Dulbecco’s modified Eagle medium (DMEM; Nacalai Tesque, Kyoto, Japan, Cat# 08458-16) supplemented with 10% FBS and 1× penicillin–streptomycin (Pe/St; Nacalai Tesque, Cat# 09367-34). VeroE6/TMPRSS2 cells (JCRB, Cat# JCRB1819) [11] were cultured in DMEM—low glucose (Sigma-Aldrich, Meguro-Ku, Japan, Cat# D6046-500ML) supplemented with 10% FBS, 20 mM HEPES (Nacalai Tesque, Cat# 17557-94), and 1 mg/mL G-418 (Nacalai Tesque, Cat# 09380-44).

### 2.3. Viruses

Influenza virus IAV (H1N1) strain A/PR/8/34 (American Type Culture Collection, Manassas, VA, USA, Cat# VR-95) was propagated in specific pathogen-free chicken embryonated eggs. Akabane virus (AKAV; TS-C2 vaccine strain) was purchased from Kyoto Biken Laboratories (Kyoto, Japan) and was propagated in HmLu-1 cells maintained in DMEM with 2% FBS and 1% Pe/St.

A pig nasal swab collected in the previous study [16] was used for viral isolation. Note that the nasal swab sample was collected from a dead animal due to natural illness and tested positive for the swine influenza virus (SIV, H1N1) using RT-PCR. The sample was inoculated into 9–11-day-old embryonated chicken eggs, which were incubated at 37 °C for 3 days. Allantoic fluid was harvested from the eggs, and commercial diagnostic influenza A immunochromatography (Fujirebio, Tokyo, Japan) was used to confirm virus isolation. All experiments were performed in a biosafety level 2 facility.

### 2.4. Determination of the Full-Length Sequence of the SIV (H1N1)

After extraction of nucleic acids from allantoic fluid, RT-PCR was performed using MBTuni-12 and -13 primers complementary to the conserved regions at both ends of the influenza A virus to amplify the full-length sequences of the eight segments of the influenza A virus [17]. Purification of RT-PCR products, library preparation, and next-generation sequencing were performed as previously described [18]. Sequences generated with next-generation sequencing were analyzed using CLC Genomics Workbench 11 software (Qiagen). Sequences were then processed to remove primers and low-quality reads and mapped to the reference genomes of SIV strain A/swine/Aichi/101/2018 (H1N1) (Acc. Num: MW269569-76) [19]. The SIV (H1N1) sequences determined in this study were submitted to the DNA Data Bank of Japan (DDBJ) under Acc. Num: LC778488-95.

### 2.5. Generation of a Retroviral Vector to Express TMPRSS2

To generate a retroviral vector expressing pig TMPRSS2, the coding sequence of pig TMPRSS2 was synthesized according to the amino acid sequences deposited in GenBank (Acc. Num: NP_001373060) with codon optimization to pig cells (Integrated DNA Technologies, Inc., Coralville, IA, USA). The synthesized DNA sequence is summarized in the S1 File. Synthesized DNA was cloned into the pDON-5 Neo-vector (TaKaRa, Kusatsu, Japan, Cat# 3657), which was prelinearized with NotI-HF (New England Biolabs (NEB), Ipswich, MA, USA, Cat# R3189L) and BamHI-HF (NEB, Cat# R3136L) using an In-Fusion HD Cloning Kit (TaKaRa, Cat# Z9633N). Plasmids were amplified using NEB 5-alpha F′ Iq competent *Escherichia coli* (NEB, Cat# C2992H) and extracted using the PureYield Plasmid Miniprep System (Promega, Madison, WI, USA, Cat# A1222). The plasmid sequence was verified using a SupreDye v3.1 Cycle Sequencing Kit (M&S TechnoSystems, Osaka, Japan, Cat# 063001) with a Spectrum Compact CE System (Promega).

### 2.6. Generation of PK-15 Cells Stably Expressing TMPRSS2

Lenti-X 293T cells were cotransfected with pDON-5 Neo-pigTMPRSS2, pGP packaging plasmid (TaKaRa, Cat# 6160), and pMD2.G plasmid with TransIT-293 Transfection Reagent (TaKaRa, Cat# V2700) in Opti-MEM (Thermo Fisher Scientific, Minoto-Ku, Japan, Cat# 31985062). The supernatant was filtered 2 days after transfection. Collected retroviral vectors were used to infect normal PK-15 or PK-15 (*Stat2* k/o) cells, which were then cultured in 500 µg/mL G-418 for six days. Single-cell cloning was then performed with a Cell Sorter SH800S (SONY, Minoto-Ku, Japan). Briefly, a single cell clone was sorted into one well of a 96-well plate. After cell growth, the expression of TMPRSS2 in each clone was evaluated with Western blotting as described below (Section 2.12).

### 2.7. Rescue of Reporter Viruses

To rescue an HIV-1–based lentiviral vector, 2.5 × 10^5^ cells Lenti-X 293T cells were cotransfected with 0.4 μg psPAX2-IN/HiBiT, 0.4 μg pWPI-Luc2, and 0.2 μg pMD2.G using 3 μL TransIT-293 Transfection Reagent in 100 μL Opti-MEM I Reduced Serum Medium. The supernatant was filtered 2 days after transfection. To measure the concentration of Gag p24 protein, the HiBiT value was measured using the Nano Glo HiBiT Lytic Detection System (Promega, Cat# N3040) as described previously [15].

### 2.8. Preparation of Standards for RT-qPCR

Standards for determining the copy number of virus stock were prepared using the PrimeScript II High Fidelity One-Step RT-PCR Kit (TaKaRa, Cat# R026A) with the primers listed in Table 1. vRNA copy numbers of each virus stock were measured using RT-qPCR assay with the One-Step TB Green PrimeScript PLUS RT-PCR Kit (Perfect Real Time) (TaKaRa, Cat# RR096A) as described below (Section 2.10). Primers used for quantification are summarized in Table 1. The PCR protocol was 42 °C for 5 min, 95 °C for 10 s, 40 cycles of 95 °C for 5 s, and 60 °C for 34 s. Levels of vRNA were normalized to those of vRNA in normal PK-15 cells (ΔΔCt method).

### 2.9. Virus Infection

Normal PK-15 and PK-15/TMPRSS2 #23 cells were seeded into a 96-well plate at 1 × 10^4^ cells per well, as previously described [14]. To test viral replication in the presence of type I interferon, cells were treated with 100 ng/mL pig IFNβ (Kingfisher Biotech, Saint Paul, MN, USA, Cat#RP0011S-025). Cells were incubated for 24 h. For IAV (H1N1) and AKAV, the culture supernatant was removed, and 100 μL per well virus solution with or without 1 μg/mL N-tosyl-L-phenylalanine chloromethyl ketone (TPCK)-treated trypsin (Sigma-Aldrich, St. Louis, MO, USA, Cat# 4352157) was added per well. After incubation at 37 °C for 2 h, the culture supernatant was removed, and the cells were washed once with DMEM + 2%FBS + Pe/St. Subsequently, 150 μL fresh DMEM + 2%FBS + Pe/St was added. The cells were treated with 50, 12.5, 3.13, 0.78, 0.2, 0.05, or 0.01 µM nafamostat mesylate (Selleckchem, Yokohama, Japan, Cat# S1386) for 2 h before infection.

For the luciferase-encoding virus, infection was performed as described above. Infected cells were lysed 2 days after infection with a britelite plus (PerkinElmer, Yokohama, Japan, Cat#6066769), and the luminescent signal was measured using a GloMax Explorer Multimode Microplate Reader (Promega).

### 2.10. Quantification of vRNA Levels in the Culture Supernatant

Normal PK-15 or PK-15/TMPRSS2 #23 cells were infected in wells in a 96-well plate (*n* = 6). After two days, the culture supernatant was collected, and virus RNA levels were measured with RT-qPCR using the One-Step TB Green PrimeScript PLUS RT-PCR Kit (Perfect Real Time) as described previously [14] using the primers summarized in Table 1. Briefly, the supernatant was mixed with 2× RNA lysis buffer (2% Triton X-100, 50 mM KCl, 100 mM Tris-HCl (pH 7.4), 40% glycerol, and 0.4 U/μL Recombinant RNase Inhibitor (TaKaRa, Cat# 2313A)) [14]. The treated supernatant was used for the RT-qPCR reaction. The PCR protocol was 42 °C for 5 min, 95 °C for 10 s, 40 cycles of 95 °C for 5 s, and 60 °C for 34 s. vRNA levels were normalized to those of the vRNA level in normal PK-15 cells, which was used as a control (ΔΔCt method).

### 2.11. Quantification of vRNA Levels in Infected Cells

Normal PK-15 and PK-15/TMPRSS2 #23 cells were infected as described above. Two days after infection, total RNA was collected using a CellAmp Direct RNA Prep Kit for RT-PCR (Real Time) (TaKaRa, Cat# 3732). GAPDH and vRNA levels were measured with an RT-qPCR assay using the One-Step TB Green PrimeScript PLUS RT-PCR Kit (Perfect Real Time). The PCR protocol was 42 °C for 5 min, 95 °C for 10 s, 40 cycles of 95 °C for 5 s, and 60 °C for 34 s. Primers used for RT-qPCR are summarized in Table 1. vRNA levels were normalized to those of porcine *Actb* mRNA, which was used as an endogenous control (ΔΔCt method).

### 2.12. Western Blotting

To evaluate TMPRSS2 expression, pelleted cells were lysed in 2× Bolt LDS sample buffer (Thermo Fisher Scientific, Cat# B0008) containing 2% β-mercaptoethanol (Bio-Rad, Hercules, CA, USA, Cat# 1610710) and incubated at 70 °C for 10 min. TMPRSS2 expression was evaluated using SimpleWestern Abby (ProteinSimple, San Jose, CA, USA) with an anti-Myc tag mouse monoclonal antibody (Cell Signaling Biotechnology, Danvers, MA, USA, Cat# 2276S, ×125) and an Anti-Mouse Detection Module (ProteinSimple, Cat# DM-001). The amount of input protein was visualized using a Total Protein Detection Module (ProteinSimple, Cat# DM-TP01). The expected size of Myc-tagged TMPRSS2 is 55.75 kDa, according to the Protein Molecular Weight website (https://www.bioinformatics.org/sms/prot_mw.html, accessed on 20 December 2023).

### 2.13. Alignment of TMPRSS2 Proteins

The MUSCLE algorithm on MEGA 11.0.13 (MEGA Software) was used to align the TMPRSS2 protein sequences from pigs (Acc. Num: NP_001373060) and humans (Acc. Num: NP_001128571.1). The alignment was visualized using CLC Genomics workbench software (version. 22.0.1, QIAGEN, Hilden, Germany).

### 2.14. Phylogenetic Analysis of Mammalian TMPRSS2

A phylogenetic tree was constructed using the TMPRSS2 sequences obtained from GenBank for various mammals including pig (Acc. Num: NP_001373060), human (Acc. Num: NP_001128571.1), cattle (Acc. Num: XP_024835315.1), horse (Acc. Num: XP_014591871.1), dog (Acc. Num: BBD33861.1), cat (Acc. Num: XP_023094478.2), mouse (Acc. Num: AAF97867.1), chimpanzee (Acc. Num: XP_001172064.5), gorilla (Acc. Num: XP_004062887.2), orangutan (Acc. Num: XP_054398540.1), bear (Acc. Num: XP_057162750.1), bat (Acc. Num: XP_037000504.2), rabbit (Acc. Num: XP_051693971.1), elephant (Acc. Num: XP_049730872.1), whale (Acc. Num: XP_007172772.2), sheep (Acc. Num: XP_042093293.1), and camel (Acc. Num: XP_031315750.1) sequences. The tree was created using the neighbor-joining method with a general time reversible nucleotide substitution model implemented in a MEGA X program [21].

### 2.15. Calculation of Identity of TMPRSS2 among Animal Species

The identity of TMPRSS2 sequences among animal species was calculated using MEGA X with a pairwise distance matrix. Analyses were conducted using the Poisson correction model. The rate variation among sites was modeled with a gamma distribution (shape parameter = 5). All ambiguous positions were removed for each sequence pair (pairwise deletion option).

### 2.16. Statistical Analysis

The results are presented as the mean and standard deviation of six measurements from one assay, representing at least two or three independent experiments. Differences in infectivity between two different conditions (e.g., between normal PK-15 and PK-15/TMPRSS2 #23 cells) were evaluated using an unpaired, two-tailed Student’s *t*-test. *p* ≤ 0.05 was considered significant. Tests were performed using Prism 9 software v9.1.1 (GraphPad, Boston, MA, USA).

## 3. Results

### 3.1. Generation and Screening of PK-15 Cells Stably Expressing Pig TMPRSS2

**Figure 1:**
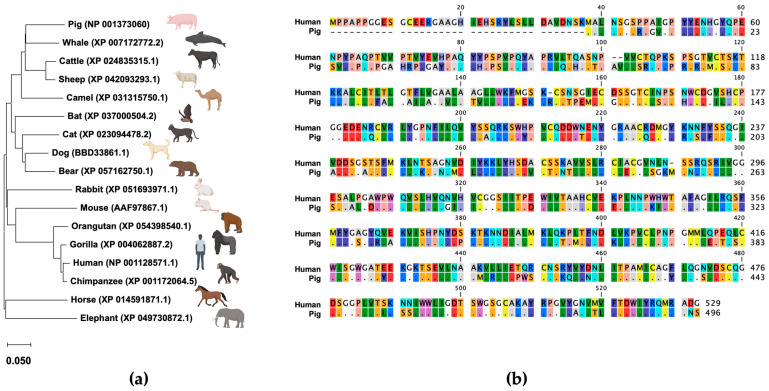
(**a**) Phylogenetic tree of TMPRSS2. A phylogenetic tree was constructed using the TMPRSS2 sequences retrieved from GenBank. The tree was constructed using the neighbor-joining method with a general time reversible nucleotide substitution model implemented in the MEGA X program [21]. (**b**) Alignment of TMPRSS2 amino acid sequences between humans and pigs.

We constructed a phylogenetic tree using the mammalian TMPRSS2 amino acid sequences retrieved from GenBank that shows the genetic difference in the TMPRSS2 sequence between humans and pigs (Figure 1a). The homology rate of TMPRSS2 sequences between humans and pigs is approximately 70%, suggesting that using cells expressing human TMPRSS2 for virus isolated from pig samples is questionable (Figure 1b).

**Figure 2:**
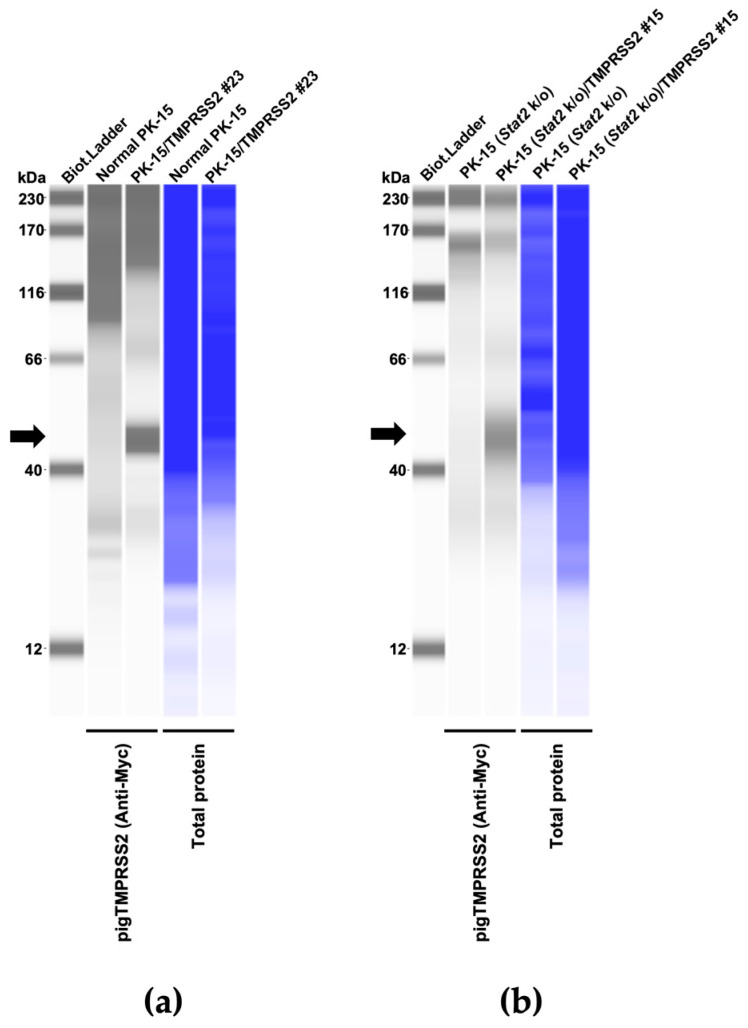
(**a**) Expression of the Myc-tagged TMPRSS2 in PK-15 cells as determined with Western blotting; the cellular lysate of normal PK-15 cells was used as a negative control (empty). (**b**) Expression of the Myc-tagged TMPRSS2 in PK-15 (*Stat2* k/o) cells as determined with Western blotting; the cellular lysate of unmodified PK-15 (*Stat2* k/o) cells was used as a negative control (empty). The black arrows indicate the size of Myc-tagged TMPRSS2.

Therefore, we aimed to generate a pig-derived cell line expressing pig TMPRSS2 (pigTMPRSS2) and designed a Myc-tagged pigTMPRSS2 using pig genomic information deposited in GenBank (Acc. Num: NP_001373060) with pig codon optimization. The synthesized DNA was cloned into a retroviral vector that was used to infect normal PK-15 cells, and the transduced cells were selected with neomycin. Single-cell clones were obtained with cell sorting, and Western blotting using an anti-Myc tag antibody was performed on each cell clone to test the expression levels of pigTMPRSS2 (Figure 2a and Supplementary Figure S1). We selected the PK-15/TMPRSS2 #23 clone because this clone had a higher expression level than that of the other clones. Furthermore, we expressed pigTMPRSS2 in PK-15 cells lacking the *Stat2* gene (PK-15 (*Stat2* k/o) cells) [14] to obtain PK-15 (*Stat2* k/o)/TMPRSS2 #15 cells (Figure 2b).

### 3.2. TMPRSS2 Expression in PK-15 Cells Enhances IAV (H1N1) Replication

**Figure 3:**
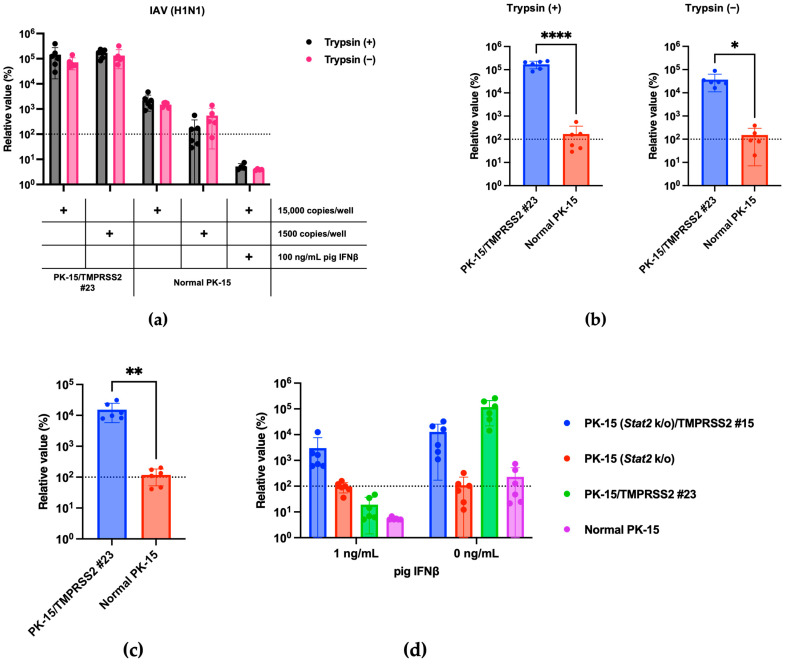
(**a**) PK-15/TMPRSS2 #23 or normal PK-15 cells were infected with 15,000 or 1500 copies of IAV (H1N1), and vRNA levels in the culture supernatant were measured with RT-qPCR 2 days later. The relative values were calculated in comparison to those in normal PK-15 cells without trypsin treatment. Cells treated with 100 ng/mL pig IFNβ served as the negative control. (**b**) The relative value was calculated according to the values in normal PK-15 cells in either the trypsin (+) or trypsin (−) treatments. Differences between PK-15/TMPRSS2 #23 and normal PK-15 cells infected with 15,000 copies of IAV (H1N1) were examined with a two-tailed, unpaired Student’s *t*-test. **** *p* < 0.0001, * *p* < 0.05. (**c**) PK-15/TMPRSS2 #23 or normal PK-15 cells were infected with 15,000 copies of IAV (H1N1), and intracellular vRNA levels were measured with RT-qPCR 2 days later. The relative values were calculated in comparison to those in normal PK-15 cells without trypsin treatment. Differences between PK-15/TMPRSS2 #23 and normal PK-15 cells were examined with a two-tailed, unpaired Student’s *t*-test. ** *p* < 0.01. (**d**) PK-15 (*Stat2* k/o)/TMPRSS2 #15, PK-15 (*Stat2* k/o), PK-15/TMPRSS2 #23, or normal PK-15 cells treated with one ng/mL pig IFNβ or left untreated for 24 h and then infected with 15,000 copies of IAV (H1N1). vRNA levels in the culture supernatant were measured with RT-qPCR 2 days after infection. The relative value was calculated according to the values in normal PK-15 cells without pig IFNβ treatment.

We hypothesized that pigTMPRSS2 expression in PK-15 cells could enhance IAV replication. To address this, we infected normal PK-15 and PK-15 cells expressing pigTMPRSS2 with the IAV (H1N1) A/PR/8/34 strain with or without TPCK-treated trypsin. In normal PK-15 cells, IAV (H1N1) replication was slightly enhanced with TPCK-treated trypsin but was significantly enhanced in PK-15/TMPRSS2 #23 cells to approximately 1000-fold that in the normal PK-15 cells. Furthermore, PK-15/TMPRSS2 #23 cells did not require TPCK-treated trypsin for IAV (H1N1) replication (Figure 3a,b). The levels of intracellular vRNA measurements also correlated with those in the supernatant (Figure 3c).

Type I interferons (IFNs) exert antiviral effects by binding to the IFN alpha and beta receptor subunit 1 (IFNAR1) on the cellular surface, and STAT2 plays a critical role in this response [13]. To avoid the inhibition of viral isolation by IFNs, we recently generated a PK-15 cell line lacking *Ifnar1* or *Stat2* genes [14]. Here, we generated PK-15 cells lacking the *Stat2* gene with stable pigTMPRSS2 (Figure 2b) and tested the usefulness of the cells for IAV (H1N1) replication in the presence of IFNs. We observed comparable replication of IAV (H1N1) in normal PK-15 and PK-15 (*Stat2* k/o) cells in the absence of pig IFNβ. We also observed enhanced IAV (H1N1) replication in PK-15/TMPRSS2 #23 and PK-15 (*Stat2* k/o)/TMPRSS2 #15 cells. However, the replication of IAV (H1N1) was significantly blocked when normal PK-15 and PK-15/TMPRSS2 #23 cells were treated with 1 ng/mL of pig IFNβ. Conversely, we observed comparable IAV (H1N1) replication in PK-15 (*Stat2* k/o) and PK-15 (*Stat2* k/o)/TMPRSS2 #15 cells compared with that in normal PK- and PK-15 (*Stat2* k/o) cells (Figure 3d). These results suggest that TMPRSS2 expression in PK-15 cells enhances IAV (H1N1) replication. Furthermore, in the presence of IFN or IFN-inducible substances in clinical samples, PK-15 (*Stat2* k/o) cells with stable TMPRSS2 expression may be useful for efficient virus isolation.

### 3.3. Enhanced SIV (H1N1) Replication in PK-15/TMPRSS2 #23 Cells

**Figure 4:**
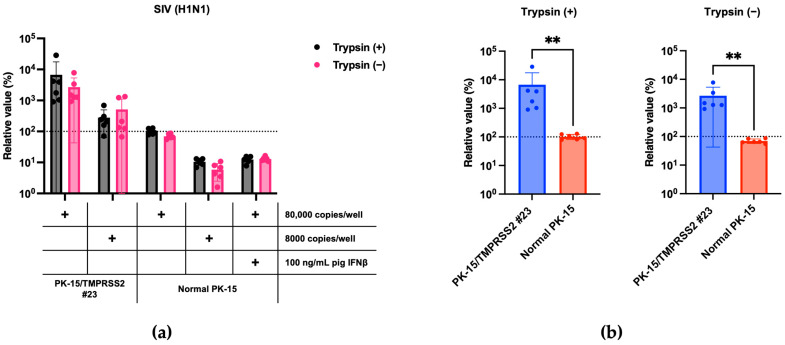
(**a**) PK-15/TMPRSS2 #23 or normal PK-15 cells were infected with 80,000 or 8000 copies of SIV (H1N1), and vRNA levels in the culture supernatant were measured with RT-qPCR 2 days later. The relative values were calculated in comparison to those in normal PK-15 cells infected with 80,000 copies of SIV (H1N1) in the absence of trypsin treatment. Cells treated with 100 ng/mL pig IFNβ served as the negative control. (**b**) The relative value was calculated according to the values in normal PK-15 cells. Differences between PK-15/TMPRSS2 #23 and normal PK-15 cells infected with 80,000 copies of SIV (H1N1) were examined with a Mann–Whitney *U*-test. ** *p* < 0.01.

Next, we tested whether the stable expression of pigTMPRSS2 promotes SIV (H1N1) replication. To address this, we infected normal PK-15 and PK-15/TMPRSS2 #23 cells with an SIV (H1N1) strain either with or without TPCK-treated trypsin. The SIV (H1N1) replication was significantly enhanced in PK-15/TMPRSS2 #23 cells to approximately 70-fold that in normal PK-15 cells. Furthermore, PK-15/TMPRSS2 #23 cells did not require TPCK-treated trypsin for SIV (H1N1) replication (Figure 4a,b). Thus, PK-15/TMPRSS2 #23 cells could be a promising tool for SIV (H1N1) propagation.

### 3.4. No Enhanced Replication of TMPRSS2-Independent Viruses in PK-15/TMPRSS2 #23 Cells

**Figure 5:**
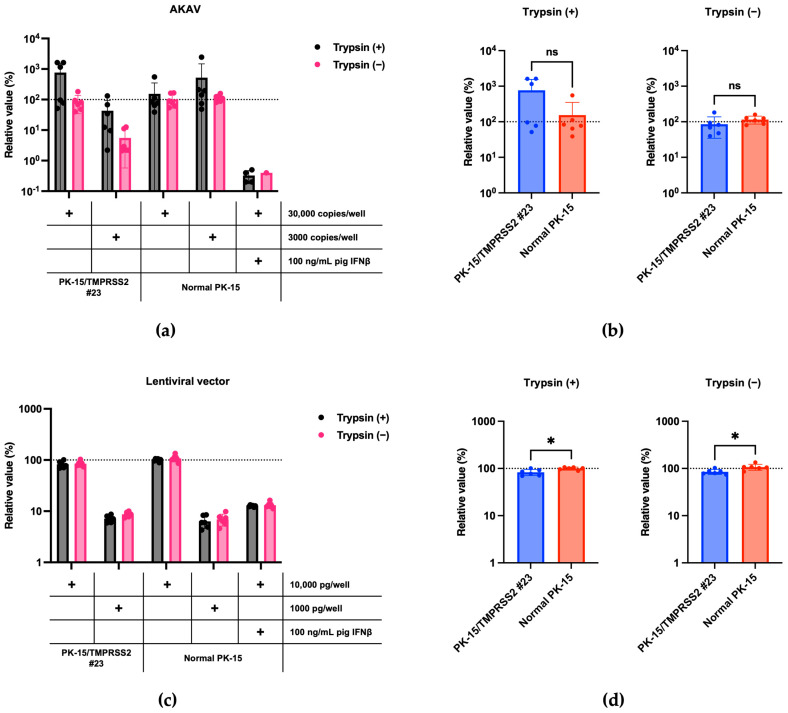
(**a**) PK-15/TMPRSS2 #23 or normal PK-15 cells were infected with 30,000 or 3000 copies of AKAV, and vRNA levels in the culture supernatant were measured with RT-qPCR 2 days later. The relative values were calculated in comparison to those in normal PK-15 cells infected with 30,000 copies of AKAV in the absence of trypsin treatment. Cells treated with 100 ng/mL pig IFNβ served as the negative control. (**b**) The relative value was calculated according to the values in normal PK-15 cells. Differences between PK-15/TMPRSS2 #23 and normal PK-15 cells infected with 30,000 copies of AKAV were examined with a two-tailed, unpaired Student’s *t*-test, ns (not significant). (**c**) PK-15/TMPRSS2 #23 or normal PK-15 cells were infected with 10,000 or 1000 pg (p24) of lentiviral vector, and luminescence was measured 2 days later. Normal PK-15 cells infected with 10,000 pg of lentiviral vector in the absence of trypsin treatment. Cells treated with 100 ng/mL pig IFNβ served as the negative control. (**d**) The relative value was calculated according to the values in normal PK-15 cells. Differences between PK-15/TMPRSS2 #23 and normal PK-15 cells infected with 10,000 pg (p24) of lentiviral vector were examined with a two-tailed, unpaired Student’s *t*-test. * *p* < 0.05.

We then investigated the specificity of an enhancement in IAV (H1N1) replication in PK-15/TMPRSS2 #23 cells. We tested whether pigTMPRSS2 expression enhanced AKAV replication since AKAV does not require TPCK-treated trypsin for viral propagation [22]. AKAV replication between normal PK-15 and PK-15/TMPRSS2 #23 cells did not significantly differ, suggesting that pigTMPRSS2 does not affect AKAV replication (Figure 5a,b). Finally, we tested whether pigTMPRSS2 enhances the infection of an HIV-1-based lentiviral vector. The lentiviral vector has a VSV-G envelope, and its infection is therefore mediated by endocytosis [23]. No significant difference was present in the lentiviral vector infection efficiency between normal PK-15 and PK-15/TMPRSS2 #23 cells. This suggests that pigTMPRSS2 expression does not affect virus replication and infection of TMPRSS2-independent viruses (Figure 5c,d). Therefore, stable pigTMPRSS2 expression in PK-15 cells promotes viral replication of viruses that require the cleavage of envelope proteins by host TMPRSS2.

### 3.5. IAV (H1N1) Replicated More Efficiently in PK-15/TMPRSS2 #23 Cells than in Vero Cells Expressing TMPRSS2

**Figure 6:**
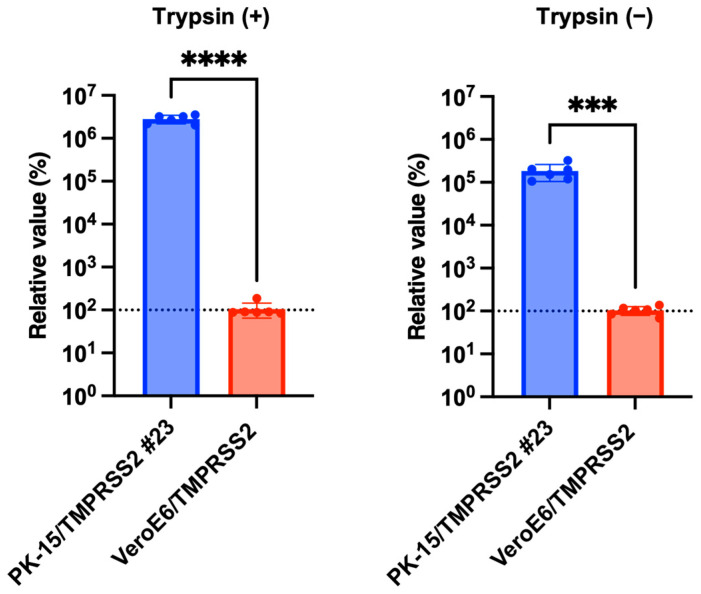
PK-15/TMPRSS2 #23 or VeroE6/TMPRSS2 cells were infected with 75,000 copies of IAV (H1N1), and vRNA levels in the culture supernatant were measured with RT-qPCR 2 days later. The relative values were calculated in comparison to that in VeroE6/TMPRSS2 cells. Differences between PK-15/TMPRSS2 #23 or VeroE6/TMPRSS2 cells were examined with a two-tailed, unpaired Student’s *t*-test. **** *p* < 0.0001, *** *p* < 0.001.

Having demonstrated that IAV (H1N1) robustly replicated in PK-15/TMPRSS2 #23 cells, we hypothesized that PK-15/TMPRSS2 #23 cells would be superior to other cell lines for IAV (H1N1) propagation. To investigate this, we compared IAV (H1N1) replication between PK-15/TMPRSS2 #23 and VeroE6/TMPRSS2 cells. IAV (H1N1) replication was significantly higher (approximately 10,000-fold) in PK-15/TMPRSS2 #23 cells than that in VeroE6/TMPRSS2 (Figure 6). Thus, PK-15/TMPRSS2 #23 cells could be a promising IAV (H1N1) propagation tool.

### 3.6. PK-15/TMPRSS2 #23 Cells Can Be Used for Testing Antiviral Drugs

**Figure 7:**
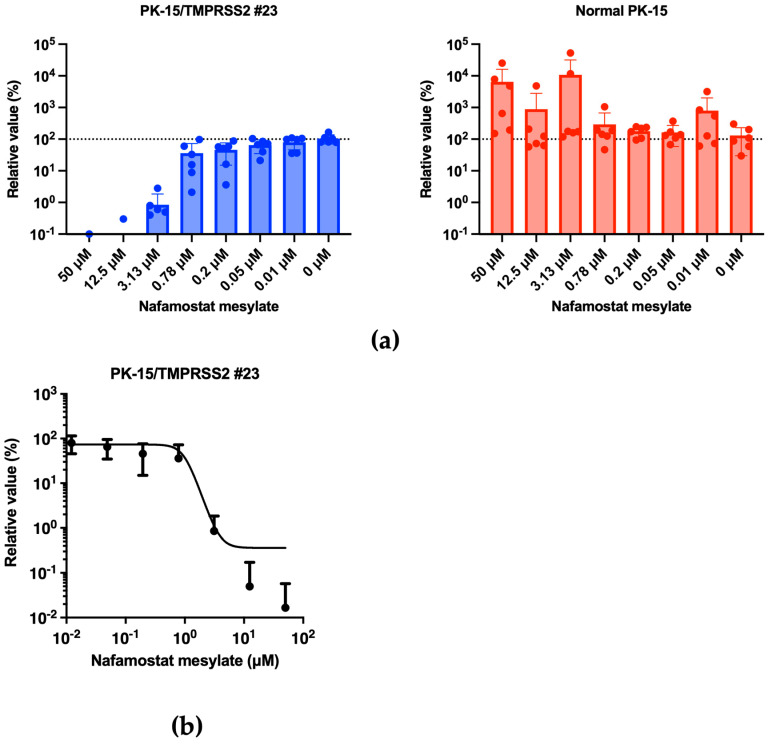
(**a**) PK-15/TMPRSS2 #23 or normal PK-15 cells were infected with 15,000 copies of IAV (H1N1) in the presence of different concentrations of nafamostat mesylate, and vRNA levels in the culture supernatant were measured were RT-qPCR 2 days later. The relative values were calculated in comparison to that in non-treated cells. (**b**) The EC_50_ of nafamostat mesylate against IAV (H1N1) in PK-15/TMPRSS2 #23 cells was calculated using Prism 9 software v9.1.1.

Previous studies showed that nafamostat mesylate explicitly inhibits the function of TMPRSS2 [24] and has consequently been used to treat SARS-CoV-2-infected individuals [25]. Therefore, we tested whether nafamostat mesylate treatment affected the enhanced IAV (H1N1) replication in PK-15/TMPRSS2 #23 cells. Infection experiments performed in the presence of different concentrations of nafamostat mesylate with normal PK-15 and PK-15/TMPRSS2 #23 cells showed that nafamostat mesylate inhibited viral replication in PK-15/TMPRSS2 #23 cells (Figure 7a). The calculated EC50 of nafamostat mesylate in PK-15/TMPRSS 2 #23 cells was 0.83651 μM. Conversely, we did not find any effect of nafamostat mesylate in normal PK-15 cells (Figure 7b).

Thus, the enhanced IAV (H1N1) replication in PK-15/TMPRSS2 #23 cells was TMPRSS2-dependent, and PK-15/TMPRSS2 #23 cells are, therefore, a valuable tool for the evaluation of inhibitors against pig TMPRSS2.

## 4. Discussion

Virus isolation from clinical specimens is essential for understanding the growth characteristics and pathogenicity of a virus strain, and an isolated virus can be used as a material to prepare a vaccine antigen. Herein, we generated a porcine PK-15 cell line that stably expressed pigTMPRSS2 to enhance the replication of TMPRSS2-dependent viruses. The PK-15/TMPRSS2 #23 cells significantly enhanced the replication of IAV (H1N1) and SIV (H1N1) even without TPCK-treated trypsin and showed marked inhibition of viral proliferation in the presence of a TMPRSS2 inhibitor. We consider that PK-15/TMPRSS2 #23 cells can improve the viral isolation efficiency of clinical samples from pigs infected with TMPRSS2-dependent viruses from respiratory or digestive organs.

TMPRSS2 plays a significant role in many respiratory viruses, including influenza viruses. Consistent with previous studies [26], the stable expression of TMPRSS2 enhanced the replication of IAV (H1N1) (Figure 3a,b) and SIV (H1N1) (Figure 4a,b). A characteristic feature of PK-15/TMPRSS2 #23 cells is that we expressed pig TMPRSS2 in pig-derived PK-15 cells. As viruses show optimal replication in their natural hosts, we believe that the PK-15/TMPRSS2 #23 cells can be an essential tool for isolating *Orthomyxoviridae* and *Coronavirinae* viruses from pigs. Furthermore, we successfully generated PK-15 (*Stat2* k/o)/TMPRSS2 #15 cells to isolate viruses in the presence of IFNs or IFN-inducible substances. To prove the usefulness of these cells, we will test the isolation efficiency of a variety of pig-derived viruses in future studies.

Interestingly, IAV (H1N1) replicated more efficiently in PK-15/TMPRSS2 #23 cells than in VeroE6/TMPRSS2 cells (Figure 6). The mechanism for this increased IAV (H1N1) replication in PK-15/TMPRSS2 #23 cells remains unclear, although the expression level of TMPRSS2 or a difference in activity between human and pig TMPRSS2 may be responsible for this phenotype. Nevertheless, PK-15/TMPRSS2 #23 cells can be a promising tool for isolating and propagating IAV (H1N1) and other TMPRSS2-dependent viruses such as porcine epidemic diarrhea virus and porcine respiratory coronavirus.

Our study has some limitations. First, one concern of gene-engineered cells is their specificity. To address this, we demonstrated that PK-15/TMPRSS2 #23 cells specifically enhanced the replications of IAV (H1N1) (Figure 3) and SIV (H1N1) (Figure 4) but not that of AKAV replication or lentiviral vector infection (Figure 5). Furthermore, we showed that nafamostat mesylate canceled the enhanced IAV (H1N1) replication in PK-15/TMPRSS2 #23 cells in a dose-dependent manner (Figure 7). Although these results demonstrated a specific enhancement in TMPRSS2-dependent viruses in PK-15/TMPRSS2 #23 cells, we need to test the applicability of PK-15/TMPRSS2 #23 cells to isolate other viruses. Second, the negative effect of stable TMPRSS2 expression on the isolation or propagation of TMPRSS2-independent viruses should be considered. In this regard, we did not observe any adverse effect of stable TMPRSS2 expression in AKAV replication or infection with lentiviral vectors (Figure 5). Nevertheless, we still need to assess this further in future studies. Third, because we used RT-qPCR to measure viral replication in this study, it is important to measure viral titers using a TCID_50_ method or a plaque assay in future studies.

In conclusion, this study established a pig-derived PK-15 cell line stably expressing pigTMPRSS2. The results clearly demonstrated that PK-15/TMPRSS2 #23 cells could replace exogenous TPCK-treated trypsin for culturing IAV (H1N1), suggesting a promising approach for isolating and propagating TMPRSS2-dependent viruses. We consider that our cells will contribute to the control of viral diseases that are prevalent in pig populations. Furthermore, these cells will be an essential tool to investigate other zoonotic viral diseases caused by TMPRSS2-dependent viruses.

## Figures and Tables

**Table 1:** Primers used in this study.

Forward	Reverse	Purpose of Use
5′-AGACAGCCACAACGGAAAAC-3′	5′-CTGTTAGGCGGGTGATGAAT-3′	Preparing a standard for IAV (H1N1) ^2^
5′-TGTTTTTGTGGGGACATCAA-3′	5′-CCCTTGGGTGTCTGACAAGT-3′	Preparing a standard for SIV (H1N1) ^2^
5′-GGGTGCGTATATGGGTCTTG-3′	5′-TCTTTGAGTGTAGCGCAGGA-3′	Preparing a standard for AKAV ^2^
5′-GGCCCAACCACAACACAAAC-3′	5′-AGCCCTCCTTCTCCGTCAGC-3′	Quantification of IAV (H1N1) vRNA ^1^
5′-TCAAGCCGGAGATAGCAATAAG-3′	5′-TTTGTCTCCCGGCTCTACTA-3′	Quantification of SIV (H1N1) vRNA ^2^
5′-GGGTTTCAGAGCCTACAAG-3′	5′-GCTACCTCAGGCAACAGATTAG-3′	Quantification of AKAV vRNA ^2^
5′-TCCCTGGAGAAGAGCTACGA-3′	5′-AGCACCGTGTTGGCGTAGAG-3′	Quantification of porcine *Actb* mRNA ^2^

^1^ Designed in the previous study [20]. ^2^ Designed in this study.

## Data Availability

The datasets of the swine influenza virus used in the current study are available in the DDBJ (Acc. Num: LC778488–LC778495).

## Supplementary Materials

The following supporting information can be downloaded at: https://www.mdpi.com/article/10.3390/pathogens13010018/s1, Figure S1: Generation of PK-15 cells stably expressing pigTMPRSS2.
