# Supplementary figures and images for: Generation of a Porcine Cell Line Stably Expressing Pig TMPRSS2 for Efficient Isolation of Swine Influenza Virus
Source: Pathogens. 2023 Dec 24;13(1):18. doi: 10.3390/pathogens13010018 (PMC10818301; doi:10.3390/pathogens13010018)

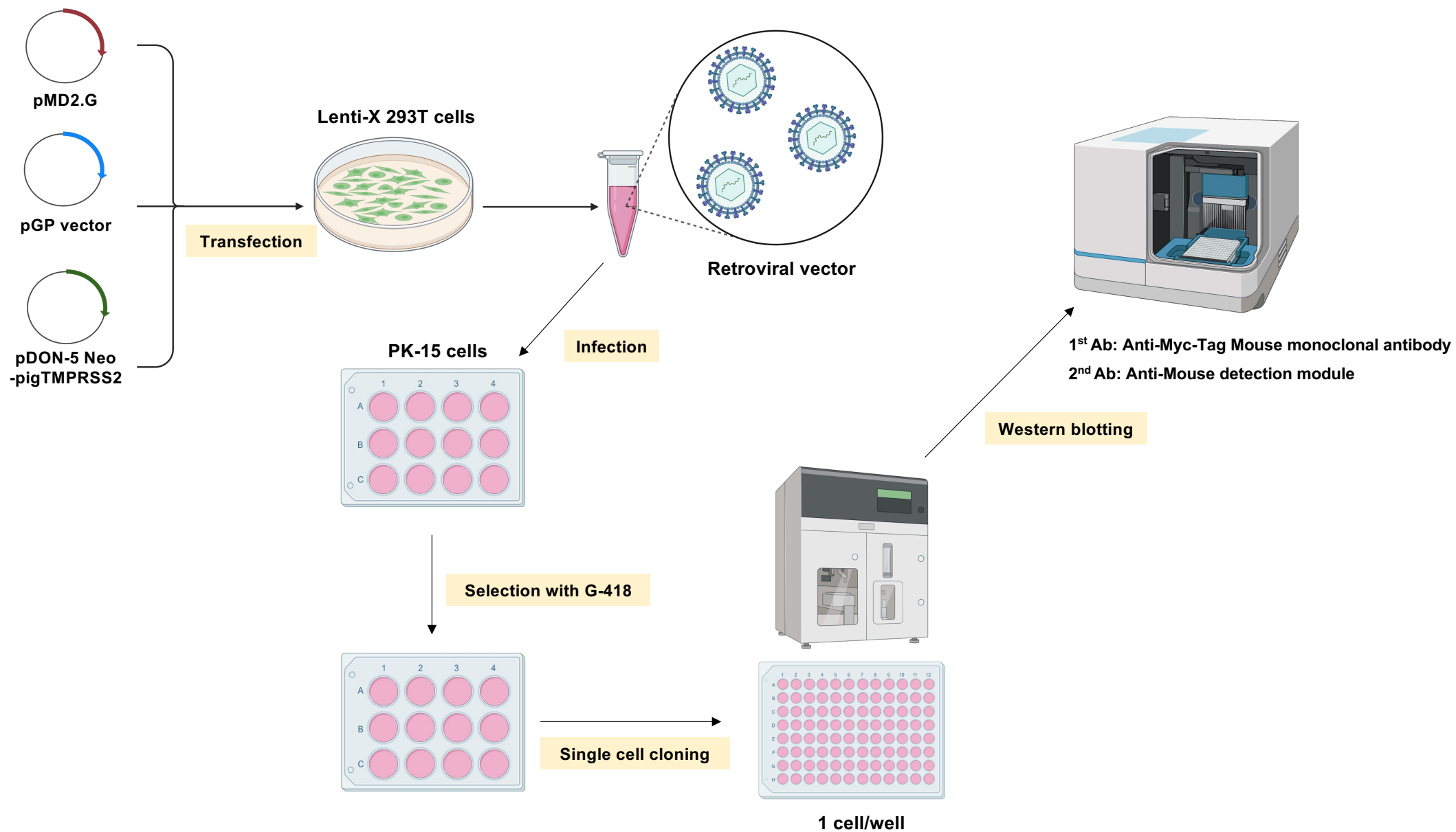

Supplement: Supplementary file 1 [file pathogens-13-00018-s001.zip › pathogens-2766913-supplementary.pdf]
